# Internet-based exercise and physical activity promotion for persons with multiple sclerosis: a randomized controlled trial

**DOI:** 10.1186/s13102-025-01146-x

**Published:** 2025-04-23

**Authors:** Verena Hartung, Alexander Tallner, Peter Flachenecker, Mathias Mäurer, René Streber, Philipp Wanner, Asarnusch Rashid, Layal Shammas, Gottfried Hois, Christian Dettmers, Holger Roick, Alexander Stefanou, Hayrettin Tumani, Susanne Weber, Klaus Pfeifer

**Affiliations:** 1 Department of Sport Science and Sport, Friedrich-Alexander-Universität Erlangen-Nürnberg, Gebbertstraße 123b, 91058 Erlangen, Germany; 2 Neurological Rehabilitation Center Quellenhof, Kuranlagenallee 2, 75323 Bad Wildbad, Germany; 3 Klinikum Würzburg Mitte gGmbH, Juliuspromenade 19, 97070 Würzburg, Germany; 4 Department of Human Movement, Training and Active Aging, Institute of Sports and Sports Sciences, Heidelberg University, Im Neuenheimer Feld 700, 69120 Heidelberg, Germany; 5 ZTM Bad Kissingen GmbH, Münchner Straße 5, 97688 Bad Kissingen, Germany; 6 medi train, Karl-Zucker-Straße, 10, 91052 Erlangen, Germany; 7 Kliniken Schmieder, Eichhornstraße 68, 78464 Konstanz, Germany; 8 E/M/S/A Center for Neurology / Psychiatry / Neuroradiology, Freiheitstraße 23, 78224 Singen, Germany; 9 Department of Neurology, Klinikum Stuttgart, Kriegsbergstraße 60, 70174 Stuttgart, Germany; 10 Specialty Hospital for Neurology Dietenbronn, Dietenbronn 7, 88477 Schwendi, Germany; 11 Department of Neurology, University Hospital Ulm, Oberer Eselsberg 45, 89081 Ulm, Germany; 12 Facharztpraxis für Neurologie und Psychiatrie, Rotebühlplatz 19, 70178 Stuttgart, Germany

**Keywords:** Multiple sclerosis, Behavior change, Exercise, Physical activity, Internet-based intervention, Physical activity-related health competence

## Abstract

**Background:**

We evaluated the effects of a 12-week internet-based exercise and physical activity promotion program for persons with multiple sclerosis.

**Methods:**

We performed a multicenter, randomized, waitlist-controlled study. The intervention group (IG) received the 12-week program, followed by 12 weeks with usual care. The control group (CG) received usual care only. The main components of the 12-week program were: (1) a tailored home-based exercise prescription, (2) e-learning resources, (3) telephone and video meetings with an exercise therapist, (4) the provision of a consumer-based PA monitor. Measurements were taken at baseline, postintervention, and after 24 weeks. The primary outcome was device-measured steps/day. Other outcomes were device-measured moderate-to-vigorous physical activity, subjectively measured leisure-time and transportation physical activity and sport/exercise, physical activity-related health competence, walking ability, quality of life, fatigue, depression, and PA-related self-concordance. We compared changes from baseline to postintervention between groups and analyzed changes in the IG during the follow-up.

**Results:**

Analysis of 56 persons with multiple sclerosis (IG: *n* = 29, CG: *n* = 27, age: 45.6 ± 10.9) revealed no significant intervention effect on steps/day. However, significant improvements were observed in moderate-to-vigorous physical activity, sport/exercise, control competence, fatigue, and quality of life (physical). During the follow-up, sport/exercise and quality of life decreased significantly. Leisure-time and transportation physical activity increased significantly.

**Conclusions:**

Our study provides first evidence that the developed program can increase control competence, aspects of physical activity and health in persons with multiple sclerosis. A trial with a larger sample is recommended to confirm our results and examine intervention mechanisms.

**Trial registration:**

Registry: Clinicaltrials.gov; registration number: NCT04367389; date of registration: 2020-04-21 (retrospectively registered).

**Supplementary Information:**

The online version contains supplementary material available at 10.1186/s13102-025-01146-x.

## Background

Multiple sclerosis (MS) affects about 2.8 million individuals worldwide [1]. It is a “chronic, inflammatory demyelinating disease of the central nervous system” [2] leading to a wide array of symptoms such as mobility limitations, spasticity, tremor, ataxia, pain, depression, fatigue, cognitive impairments, bladder and bowel problems, sensory impairments and vision loss [3, 4].

Substantial evidence confirms manifold positive effects of exercise in persons with MS (pwMS), including improvements of MS symptoms like fatigue, depression and walking ability as well as aerobic and muscular fitness, balance, and quality of life [5–11]. Furthermore, exercise does not appear to increase the risk of adverse events or relapses [12, 13]. At the same time, pwMS are generally more physically inactive than healthy controls [14, 15]. Thus, interventions are needed that promote physical activity (PA) and exercise among pwMS.

Known barriers of PA and exercise among pwMS are the availability and accessibility of PA programs and opportunities [16, 17]. A lack of PA and exercise opportunities and disabled facilities can hinder pwMS to participate in PA and exercise [16, 17]. Limited regular and disabled parking, accessibility within facilities, costs [16, 17], time, long travel distances [16], and inflexible public transport [17], may restrict access to PA and exercise opportunities. Furthermore, some pwMS are reliant on mobility aid and home adaptations, which can be a barrier to engagement in PA [17]. Internet-based remote interventions can bypass those barriers and have been shown to be feasible and safe for pwMS [18–20]. To this end, we developed and evaluated an internet-based exercise and PA promotion program for pwMS.

Based on current evidence, it is recommended to incorporate appropriate device-based methods of PA measurement in research [21]. Daily steps can be measured with pedometers and activity monitors [21] and represent an easy-to-interpret metric for overall PA [22]. Therefore, the primary objective of this study was to evaluate the effectiveness of a 12-week internet-based exercise and PA promotion program on device-measured daily steps of pwMS. The secondary objective was to evaluate the effects on the volume of (a) device-measured minutes of moderate-to-vigorous intensity PA (MVPA), (b) self-reported minutes of sport- and exercise-related PA, (c) self-reported minutes of leisure-time/ transportation PA, and (d) PA-related health competence (PAHCO; [23, 24]) as a measure of personal prerequisites for a self-regulated, habitual as well as health-enhancing physically active lifestyle. Furthermore, we evaluated the effects of the program on fatigue, quality of life, mobility, and PA-related and sports-related self-concordance as well as the usability and usefulness of the mobile application. An additional exploratory analysis aimed to evaluate the effects after a 12-week follow-up phase.

## Methods

We conducted a multicenter, randomized, waitlist-controlled study from October 2019 to June 2020. This study was part of the project “MS bewegt” [engl. MS moves]. The “MS bewegt” project aimed to develop and evaluate an internet-based competence-oriented intervention for the promotion of PA and exercise. Ethical approval was granted by the ethics committee of the Baden-Württemberg Federal Chamber of Physicians (Sign F-2018-059). The study was registered on *ClinicalTrials.gov* (NCT04367389; retrospectively registered; registration date: 24/04/2020) and its reporting follows the CONSORT guidelines for parallel group randomized trials [25].

Participants were recruited through leaflets, phone calls, or medical consultation hours in eight study centers (three rehabilitation centers, two hospitals and three resident neurologists) located in the south of Germany from October to November 2019. PwMS that showed interest to participate in the study received written information about the study including study aims, contents and procedures as well as the voluntariness of study participation. Additionally, they attended an informational meeting with study personnel of the respective study site. During this meeting, pwMS were informed about the study and open questions were clarified. All enrolled participants provided their voluntary and informed written consent to participate in the study.

The inclusion criteria were: (1) age ≥ 18 years, (2) MS diagnosis according to the McDonald criteria [26], (3) Expanded Disability Status Scale (EDSS) score of 0–6.5 [27], (4) no exacerbation within 30 days prior to enrolment, (5) wireless internet access, (6) basic computer and internet skills, (7) smartphone possession (Android or IOS), (8) ability to operate a smartphone (especially to install mobile applications), (9) ability to read, write and comprehend as well as communicate electronically. Patients were excluded if they (1) were exercising regularly (more than two times per week for at least 30 min with moderate or high intensity), (2) received corticosteroid therapy within the last 30 days, (3) had a clinically relevant cardiovascular disease, (4) cognitive impairments or severe impairment of hand function that hampered study participation, or (5) severe internal, orthopedic and metabolic comorbidities that restrict mobility.

After the study centers had enrolled the study participants, one researcher (A. T.) assigned participants equally into the intervention (IG) or waitlist control group (CG) using a stratified permuted block randomization. The strata were disability level (measured with the EDSS) and PA level (operationalized as daily steps), because evidence suggests that both can influence the response to the intervention under investigation as well as overall prognosis [28–30]. Within each stratification block, we used a block randomization with blocks of four to randomly assign participants to one of the two groups. The list with the random allocation sequence was generated with Excel (Microsoft Cooperation, Redmond, Washington, USA). Study centers were not informed about the group allocation of participants. The IG received the 12-week internet-based program, followed by 12 weeks with usual care and no additional intervention. The CG received usual care during the first 12-weeks followed by the 12-week intervention. Measurements were taken at baseline prior to randomization (T0), after 12 weeks (T1; immediately after the intervention in the IG) and after 24 weeks (T2).

### Intervention

We developed a 12-week internet-based exercise and PA promotion intervention. Theoretically based on the PAHCO model [23, 24] and self-determination theory (SDT) [31], the intervention was designed to improve competences for a healthy, physically active lifestyle and thus to promote health-enhancing PA.

According to the PAHCO model, individuals require three subcompetences to pursue a healthy, physically active lifestyle: movement competence, control competence, and self-regulation competence [23, 32]. A certain level of movement competence is needed to adequately meet the immediate motor-related demands of health-related activities (e.g. walking, cycling, body weight exercises for muscle strengthening) and master challenges of daily life (e.g. lifting loads, climbing the stairs). Self-regulation competence constitutes the motivational and volitional basis of regular PA. Finally, an appropriate level of control competence is needed to align the physical load of PA with positive effects on health and well-being. These three subcompetences in turn emerge from the integration of specific dispositions of PA. A detailed description of the PAHCO model can be found elsewhere [23, 32]. The PAHCO model furthermore specifies that exercise and physical practice, learning, and experiencing of individuals need to be systematically linked to one another for competences to develop. Supplement 1 provides an overview of the intervention content that was used to promote the three subcompetences of the PAHCO model.

The SDT provided additional guidelines for the intervention design. The SDT describes the motivational prerequisites of a behavior. It distinguishes between amotivation, extrinsic motivation and intrinsic motivation [33]. While intrinsically motivated behavior is always self-determined, extrinsically motivated behavior can be either internally regulated, thus self-determined, or externally regulated. The more self-determined an extrinsically motivated behavior is, the higher the expected performance, persistence and the better the positive experience related to that behavior [33]. According to the SDT the three basic needs (competence, autonomy, and relatedness) need to be satisfied during activities in order to promote self-determined behavior [34]. We used the key component techniques for need-support described by Silva et al. [35] to foster self-determined PA. Supplement 2 provides information about the implementation of these key component techniques in the developed program.

#### Intervention components

The intervention comprised (1) a tailored home-based exercise prescription, (2) e-learning resources, (3) telephone and video meetings with the therapist, and (4) the provision of a consumer-based PA monitor. This chapter provides further information about the individual intervention components. A detailed example of a program has been published previously as part of a case study [36]. Three certified exercise therapists delivered the intervention using a Microsoft Azure (Microsoft Corporation, Redmond, WA, USA) cloud computing platform (customized by proMX AG, Nuremberg, Germany; provided by motionNET systems Ltd., Nuremberg, Germany), which served as their control center for intervention delivery. Each participant was supervised and coached one-on-one by a therapist, apart from the two group video calls (described later). The tailored home-based exercise prescription was provided with an app for study participants, available as a smartphone and web application (Zentrum für Telemedizin Bad Kissingen GmbH, Bad Kissingen, Germany). Furthermore, the application granted participants access to their personalized goals (weekly training sessions, PA minutes and daily steps), an overview of their daily and weekly progress and a PA diary. Participants had the option to chat with their therapist and analyze their training progress throughout the entire intervention period. E-learning resources were created and made accessible with ILIAS (ILIAS open source e-learning e. V., Cologne, Germany), which is an open-source web-based learning management system.

##### Tailored home-based exercises

Based on participants’ needs, physical capacity and personal exercise equipment, therapists agreed on a personalized home-based exercise regime with each participant. Therapists were instructed to prescribe endurance and resistance training in line with PA guidelines for persons with MS [37, 38]. Endurance exercises were recommended 1–2 times per week for 10–60 min at a rate of perceived exertion (RPE; [39]) of 11–15 (i.e. light to moderate intensity). Participants and therapists chose the type of endurance activity collaboratively. Resistance training, containing 6–8 exercises targeting major muscle groups, was recommended 1–2 times per week (8–20 repetitions, 2–3 sets, RPE of 11–15). Exercises could be performed indoors and outdoors. Exercise dose was progressed semi-automatically, based on participants’ RPE and predetermined progression paths. Whenever a participant reported an RPE above the targeted range or experienced pain after an exercise, the therapist had to review and adapt the respective exercise plan. Therapists compiled exercise plans using a wide selection of strength exercises and endurance activities available on the cloud computing platform. Participants received access to their exercise prescription through the study app. In the study app, participants were guided through each exercise session. Instructions were presented for each exercise through text, photos and a prescription of repetitions and sets. After each exercise, participants were prompted to rate exercise intensity based on RPE.

##### E-learning

Patients were offered 17 learning modules in the four subject areas “Technology in MS bewegt” (four modules), “Exercise and Symptoms of MS” (three modules), “Volitional and motivational prerequisites of physical activity” (five modules) and “How to plan and monitor exercise” (five modules). A list of the single modules is provided in Supplement 3. The four modules of the subject area “Technology in MS bewegt” were made accessible prior to the first call between participant and therapist to support participants in setting up the technical components of the intervention (app, video calls, activity monitor). The activation of the remaining 13 modules by the therapists was optional, as it was assumed that the participants would have varying needs regarding the promotion of a physically active, healthy lifestyle due to differences in MS symptoms and PAHCO levels. The therapists were able to grant access to a flexible number of modules after each telephone or video meeting. During the meetings, therapists and participants agreed on the activation of specific modules based on participant’s individual needs with regard to the regular, long-term engagement in health-enhancing PA, their interests and their time available. Each module was developed to have a workload of 10 to 20 min.

##### Telephone and video meetings

The web- and telephone-based coaching consisted of two one-on-one telephone or video calls (weeks 1 and 7) and two group video calls with two to four participants (weeks 4 and 11). During the 60-minute one-on-one call in week 1, participants and therapists talked about the participant’s medical and PA history and agreed on personalized learning, exercise, and step goals. In week 4, participants shared their progress and experiences with one another during a 45-minute group video call. In week 7, participants reflected on the first part of the intervention and goal achievement together with their therapists in a 20-minute one-on-one call. If goals were not achieved, reasons were identified. If necessary, learning and exercise goals were adjusted. During the group video call in week 11 (45 min), participants reflected their individual goal attainment, shared lessons learned and PA plans for the time after the program. Therapists’ communication strategies during telephone and group video calls were informed by motivational interviewing [40]. Therapists received a two-day motivational interviewing training prior to study start.

##### Activity monitors

Each participant received a consumer-grade PA monitor at the beginning of the intervention to enable self-monitoring of daily steps. Patients using walking aids received the Fitbit Inspire (Fitbit Inc., San Francisco, CA, USA) and were instructed to wear it on the hip. The remaining patients received the wrist-worn Garmin vivofit 4 (Garmin Ltd., Olathe, KS, USA). Patients were advised to wear the PA monitor every day throughout the 12-week intervention. Step measurement of both consumer-grade PA monitors has been validated for pwMS during over-ground walking [41]. Daily steps were automatically imported to the telemedicine platform and the study app via a Bluetooth connection.

### Measures

Age, gender, disease severity (EDSS), disease duration, and the use of physiotherapy treatment (yes/no) were collected at baseline. We planned to measure all outcomes at baseline, after 12 weeks and 24 weeks. However, clinical assessments in the study centers (2-Minute Walk Test and Timed 25-Foot Walk) could not be performed after 24 weeks since the time of measurement coincided with the COVID-19 lockdown in Germany. Participants completed questionnaires and underwent clinical tests (2-Minute Walk Test and Timed 25-Foot Walk) at the study centers at baseline and after 12 weeks. After 24 weeks, the researchers from the Friedrich-Alexander-Universität Erlangen-Nürnberg sent the questionnaires to participants due to the COVID-19 lockdown.

#### Primary outcome

Steps per day were measured with ActiGraph model wGT3X-BT accelerometers (ActiGraph LLC, Pensacola, FL, USA). The device was mailed to participants by the researchers of the Friedrich-Alexander-Universität Erlangen-Nürnberg. Participants wore the ActiGraph on the left hip, above the anterior superior iliac spine. We instructed participants to wear the device for seven consecutive days during waking hours and to remove it only for swimming and bathing. Additionally, participants were asked to complete a diary indicating wear times. We used the software ActiLife (version 6.13.4; ActiGraph LLC, Pensacola, FL, USA) to initialize data collection as well as download and analyze data. Data were collected at 100 Hz and converted to 15-s epochs using the regular ActiLife filter. Non-wear time was determined as suggested by Choi et al. [42]. Only valid data from participants were included in the analysis, that is datasets with at least four days with a minimum of ten hours of wear time per day.

#### Secondary outcomes

##### Device-measured MVPA

The weekly minutes of MVPA were calculated based on the collected ActiGraph data using the software ActiLife. To identify MVPA, cut-off points for pwMS introduced by Sandroff et al. [43] for mild/moderate (0 ≤ EDSS ≤ 5.5) and severe disability (EDSS ≥ 6.0) were used. In accordance with the procedure applied by Sandroff et al. [43], data were converted to 15-second epochs applying ActiGraph’s low frequency extension filter prior to applying the MS-specific cut-off points. The definition of wear-time and valid data were in accordance with the data processing to determine steps per day.

##### Self-reported PA

Additionally, PA was measured subjectively with the Physical Activity, Exercise and Sport Questionnaire (BSA) [44] using a recall period of two weeks. The questionnaire asks for the frequency and duration of leisure-time/transportation PA (eight dimensions) and sport-/exercise-related activities (up to three free specifications) during a typical week. We calculated the leisure-time/transportation index and the sport and exercise index. Both report the volume of PA in minutes per week.

##### PAHCO

PAHCO was measured with a self-administered, 42-item questionnaire that has been validated for pwMS [24]. The items can be assigned to ten first-order scales representing the basic elements of PAHCO, which form the basis for the calculation of sum scores for the three subcompetences of PAHCO: movement competence, control competence, self-regulation competence. Scores for each subcompetence were calculated as percentages. Higher scores indicate higher levels of competence.

#### Other outcomes

##### Walking ability

We assessed walking speed and lower extremity functionality with the Timed 25-Foot Walk (T25FW) [45]. Walking endurance was measured with the Two-Minute Walk Test (2MWT) [46] and subjective walking ability with the German version of the Multiple Sclerosis Walking Scale (MSWS-12/D) [47]. The MSWS-12 is a 12-item questionnaire with a total score between 0 and 100. Higher scores indicate a greater impact of MS on walking.

##### Quality of life

We measured quality of life and limitations due to MS with the German version of the Multiple Sclerosis Impact Scale (MSIS-29) [48] on two subscales (physical impact of MS, 20 items; psychological impact of MS, 9 items). On an overall scale from 0 to 100, higher scores represent worse health.

##### Fatigue

The Würzburg Fatigue Inventory in MS (WEIMuS) contains 17 items that form a physical and cognitive subscale [49]. The total score ranges from zero to 68 points (maximum fatigue), with a cut-off value of 32 indicating the presence of fatigue [50].

##### Depression

A German version of the Center for Epidemiologic Studies Depression Scale (CES-D) was used to measure depression [51]. It is a 20-item questionnaire with a total score that ranges from 0 to 60. Higher scores represent higher levels of depression.

##### Physical activity-related and sports-related self-concordance

The SSK-Scale measures the extent to which the intention to exercise regularly is aligned with personal interests or values [52]. The scale was used due to its theoretical reference to the SDT. In contrast to the behavioral regulation styles in SDT (intrinsic, identified, introjected, integrated and extrinsic regulation), self-concordance can also be measured before the target behavior is performed [52] and thus, can be used for baseline measurements in physically inactive samples. The SSK-Scale contains 12 items which are assigned to four subscales: intrinsic, identified, introjected and extrinsic motivation. An overall score can be calculated based on these four subscales (minimal score: -10, maximal Score: +10). A high score suggests a high level of PA-related and sports-related self-concordance [52].

##### Usability and usefulness of the mobile application

We used module I of the meCUE questionnaire 2.0 to assess the usability and usefulness of the mobile application [53]. The module contains three items on usability and three items on usefulness that are answered on a 7-point Likert scale (scored from 1 to 7). Scores for usability and usefulness are obtained by averaging the items of each area [53].

##### Adverse events

Adverse events were recorded retrospectively as part of the assessments in the study centers after 12 weeks. Furthermore, study participants were asked to inform their study center as soon as an adverse event occurs. If participants reported an adverse event to their exercise therapist, the exercise therapist reported the adverse event to the study management. Furthermore, therapists asked the participants to consult a physician at a study center if necessary.

#### Intervention adherence and chat function usage in the app

Completed training sessions, e-learning modules of the subject areas “Exercise and Symptoms of MS”, “Volitional and motivational prerequisites of physical activity”, and “How to plan and monitor exercise” (13 modules) as well as telephone and video meetings were electronically documented with the study app. Furthermore, the number of chat messages sent between participants and therapists were documented. We calculated the average training sessions per week, the average number of modules assigned and completed, the percentage of completed meetings, the average number of chat messages per week, and the percentage of participants that used the chat function.

### Statistical analysis

We used the software G*Power [54] to perform an a priori power analysis to estimate the appropriate sample size for detecting a condition (between-subjects factor: intervention vs. control) x time (within-subjects factor: baseline, 12 weeks) interaction on the primary outcome (daily steps) with a 2 × 2 mixed analysis of variance (ANOVA). For the identification of a small to medium effect (f = 0.2) with a statistical power of 80% and a significance level of *p* ≤.05, results indicated that 52 subjects (*n* = 26 per group) are needed. The effect size for the sample size calculation was determined based on observed effect sizes from previous RCTs that reported the effects of digital PA promotion interventions and employed objective measures of PA [55, 56]. With an expected drop-out of about 25%, we aimed at recruiting 70 pwMS.

Imputation and analyses were performed with the software R, version 4.3.1 [57], using the packages *coin* (Mann-Whitney U tests) [58], *stats* (Wilcoxon signed rank test, Welsh’s t-test) [57], and *gmodels* (Fishers’s exact test) [59]. Graphs were created with the R package *ggplot2* [60]. To identify baseline differences between groups, we used the Welsh’s t-test for age, disease duration, and outcome variables, the Wilcoxon rank-sum test for the EDSS, and the Fisher’s exact test for nominal data. We performed an intention-to-treat analysis for the primary and secondary outcomes. We imputed missing values of primary and secondary outcomes for data at baseline, 12 weeks and 24 weeks. Missing values were imputed with mean group changes from the baseline to the 12-week measurement and the 12-week to the 24-week measurement. For the primary and secondary outcomes, sensitivity analyses were performed using all available cases for each analysis (pairwise deletion).

The Shapiro–Wilk test indicated that normal distribution was not present in the majority of analyzed variables. Hence, nonparametric tests were used instead of ANOVAs for all comparisons. To evaluate immediate intervention effects on measured outcomes, we used Mann-Whitney U tests to compare changes from baseline to the 12-week measurement between the IG and CG. Within-group effects from baseline to the 12-week measurement were evaluated with the Wilcoxon signed-rank test for both groups. To evaluate the sustainability of effects, changes from the 12-week measurement (postintervention) to the 24-week measurement within the IG were analyzed with the Wilcoxon signed-rank test. Effect sizes for the Mann-Whitney U tests and the Wilcoxon signed-rank tests were calculated as the correlation coefficient *r* and converted to Cohen’s *d* [61]. Cohen’s *d* effect sizes are interpreted as small, moderate, or large based on values of 0.2, 0.5, and 0.8, respectively.

## Results

**Fig. 1:**
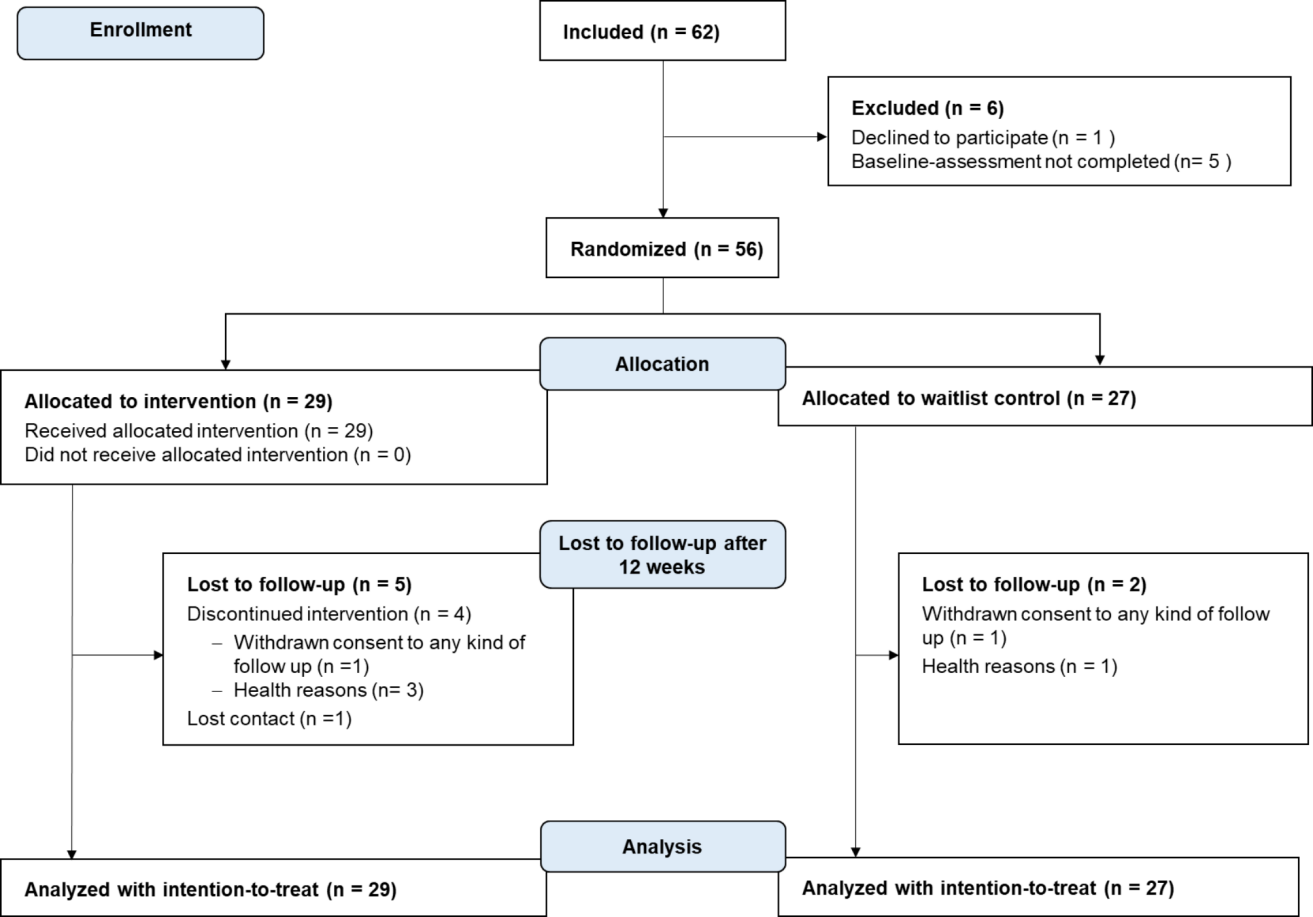
Consort flow-chart for the primary outcome

We enrolled 62 pwMS in the study. Our aim to recruit 70 pwMS could not be fully achieved since average recruitment rates in the study centers were lower than expected. Out of the 62 enrolled pwMS, 56 pwMS were randomized into the IG and a CG. Five participants of the IG and two participants of the CG were lost to follow-up (Fig. 1). The last measurement was collected at the end of July 2020. The percentages of missing values varied between 0 and 30.4% on the item level. In total, 45 out of 56 records (80.4%) were incomplete. For the primary outcome steps per day, 48 complete records were available for the baseline and 12-week measurement. Outliers were retained in the analysis, as the inspection of extreme values did not provide any evidence to suggest that they were not representative of the population under investigation [62].

Baseline values of sample characteristics (Table 1) did not differ significantly between the two groups. However, the difference between IG and CG may be clinically meaningful for fatigue. While the median value of the WEIMuS was below the cut-off value of fatigue (WEIMuS > 32) in the CG, it was well above this value in the IG.

Results of the Mann-Whitney U tests are reported in Table 2 (comparison of changes from baseline to the 12-week measurement between the IG and CG). The results of the sensitivity analyses with all available cases are reported in supplement 4. Within-group changes from baseline to the 12-week measurement and results of the Wilcoxon signed-rank tests are reported in supplemental material 5. Changes in the IG from the 12-week measurement (postintervention) to the 24-week measurement and results of the Wilcoxon signed-rank tests are reported in Table 3.

**Table 1:** Demographics and baseline characteristics of the intervention and control group

	*n*	Intervention group	*n*	Control group	*p*-value
**Age** [years], mean ± SD	29	45.4 ± 11.6	27	45.7 ± 10.4	0.922*
**Women**, n (%)	29	21 (72.4)	27	21 (77.8)	0.761***
**Disease duration** [years], mean ± SD	29	9.0 ± 7.5	27	11.1 ± 8.0	0.310*
**EDSS**, median (IQR)	29	3.5 (2.0)	27	3.0 (3.5)	0.987**
**RRMS/SPMS/PPMS**, n (%)	29	22/4/3 (75.9/13.8/10.3)	27	18/6/3 (66.7/22.2/11.1)	0.761***
**Regular physiotherapy**, n **(**%)	28	16 (57.1)	27	12 (44.4)	0.423***
**Steps per day**, median (IQR)	29	5086.4 (2179.9)	27	4907.3 (2744.4)	0.996*
**MVPA** [minutes/week], median (IQR)	29	27.5 (18.4)	27	29.9 (17.8)	0.484*
**Leisure-time/transportation PA** [minutes/week], median (IQR)	29	260.0 (485.9)	27	170.0 (432.3)	0.111*
**Sport/exercise** [minutes/week], median (IQR)	29	0.0 (49.6)	27	0.0 (42.5)	0.883*
**Movement Competence**, median (IQR)	29	50.0 (22.5)	27	44.2 (43.3)	0.718*
**Control Competence**, median (IQR)	29	39.9 (21.1)	27	52.4 (25.6)	0.276*
**Self-Regulation Competence**, median (IQR)	29	64.8 (26.8)	27	66.9 (18.1)	0.574*
**SSK-Index**, median (IQR)	28	3.0 (3.0)	27	4.0 (3.0)	0.224*
**WEIMuS**, median (IQR)	29	41.0 (30.0)	25	27.0 (31.0)	0.221*
**CES-D**, median (IQR)	26	16.5 (9.0)	25	16.0 (18.0)	0.921*
**MSIS-29 (physical)**, median (IQR)	29	35.0 (23.8)	26	33.1 (41.6)	0.908*
**MSIS-29 (psychological)**, median (IQR)	29	30.6 (24.3)	26	22.2 (36.8)	0.880*
**2minWT**, median (IQR)	27	152.4 (78.4)	27	145.0 (58.8)	0.777*
**T25FW**, median (IQR)	28	5.3 (1.9)	27	6.1 (2.0)	0.411*
**MSWS-12**, median (IQR)	28	41.7 (57.8)	27	50.0 (56.3)	0.389*

Note. *calculated with the Welsh’s t-test, **calculated with the Wilcoxon rank-sum test, ***calculated with the Fisher’s exact test; Abbreviations. 2MWT: Two-Minute Walk Test; CES-D: Center for Epidemiologic Studies Depression Scale; EDSS: Expanded Disability Status Scale; IQR: interquartile range; MSIS-29: Multiple Sclerosis Impact Scale; MSWS-12: Multiple Sclerosis Walking Scale; MVPA: moderate-to-vigorous intensity physical activity; PA: physical activity; PMMS: primary-progressive multiple sclerosis; RRMS: Relapsing-remitting multiple sclerosis; SD: standard deviation; SPMS: secondary-progressive multiple sclerosis; SSK: sports-related self-concordance; T25FW: Timed 25-Foot Walk; WEIMuS: Würzburg Fatigue Inventory in MS

**Table 2:** Median and mean differences between baseline (T0) and after 12 weeks (T1) and results of the Mann-Whitney-U-Test

Dependent variables	Intervention group			Control Group			Test-Statistics		
	*n*	Median change_T1−T0_ (IQR)	Mean change_T1−T0_ (SD)	*n*	Median change _T1−T0_ (IQR)	Mean change_T1−T0_ (SD)	Z	*p*	ES (d^a^)
Steps per day	29	-16.5 (1294.9)	-16.5 (1306.6)	27	-485.8 (1224.0)	-431.8 (1369.3)	1.550	0.123	0.42
MVPA [minutes/day]	29	1.8 (3.6)	1.8 (13.0)	27	-4.9 (11.2)	-3.7 (12.8)	2.075	0.038	0.58
Leisure-time/transportation PA [minutes/week]	29	-39.1 (39.1)	-38.7 (180.0)	27	-42.9 (67.5)	-41.4 (480.5)	1.553	0.122	0.42
Sport and exercise [minutes/week]	29	98.5 (136.3)	98.5 (157.1)	27	10.0 (57.4)	54.7 (171.4)	2.204	0.027	0.62
Movement competence [%]	29	-0.3 (15.1)	-0.3 (13.9)	27	-1.3 (9.6)	-0.9 (8.5)	0.451	0.657	0.12
Control competence [%]	29	11.4 (16.6)	11.4 (16.0)	27	0.3 (16.2)	0.3 (15.2)	2.960	0.003	0.86
Self-regulation Competence [%]	29	3.8 (10.4)	3.8 (13.6)	27	0.00 (13.7)	-0.7 (12.2)	0.919	0.363	0.25
SSK-Index	24	0.2 (3.5)	-0.1 (2.4)	26	-0.8 (1.7)	-0.8 (1.4)	1.555	0.122	0.45
2MWT	20	6.0 (23.9)	11.5 (30.4)	23	-1.0 (24.8)	1.6 (25.9)	0.585	0.567	0.18
T25FW	20	-0.4 (1.4)	-3.1 (11.7)	23	0.0 (0.7)	0.2 (1.7)	-1.620	0.107	-0.51
MSWS-12	23	-8.3 (15.6)	-8.8 (16.7)	25	-6.3 (10.4)	-3.9 (10.4)	-1.034	0.307	-0.3
WEIMuS	22	-8.5 (12.5)	-9.7 (13.6)	23	-4.0 (10.5)	-2.0 (9.4)	-2.205	0.027	-0.7
CES-D	17	0.0 (4.00)	-1.8 (6.0)	20	-1.0 (8.3)	1.8 (7.6)	-0.858	0.400	-0.28
MSIS-29, physical subscale	24	-5.4 (18.1)	-7.2 (14.4)	23	0.0 (12.5)	1.4 (11.8)	-1.971	0.049	-0.6
MSIS-29, psychological subscale	24	-5.6 (18.1)	-6.9 (20.2)	23	0.0 (15.3)	-0.1 (16.9)	-1.416	0.160	-0.42

Notes. ^*a*^Cohen’s *d* (values around 0.2 represent small effects, values around 0.5 intermediate effects, and values around 0.8 strong effects). Abbreviations: 2MWT: Two-Minute Walk Test; CES-D: Center for Epidemiologic Studies Depression Scale; IQR: interquartile range; MSIS-29: Multiple Sclerosis Impact Scale; MSWS-12: Multiple Sclerosis Walking Scale; MVPA: moderate-to-vigorous intensity physical activity; PA: physical activity; SD: standard deviation; SSK: sports-related self-concordance; T25FW: Timed 25-Foot Walk; WEIMuS: Würzburg Fatigue Inventory in MS

### Physical activity outcomes

**Fig. 2:**
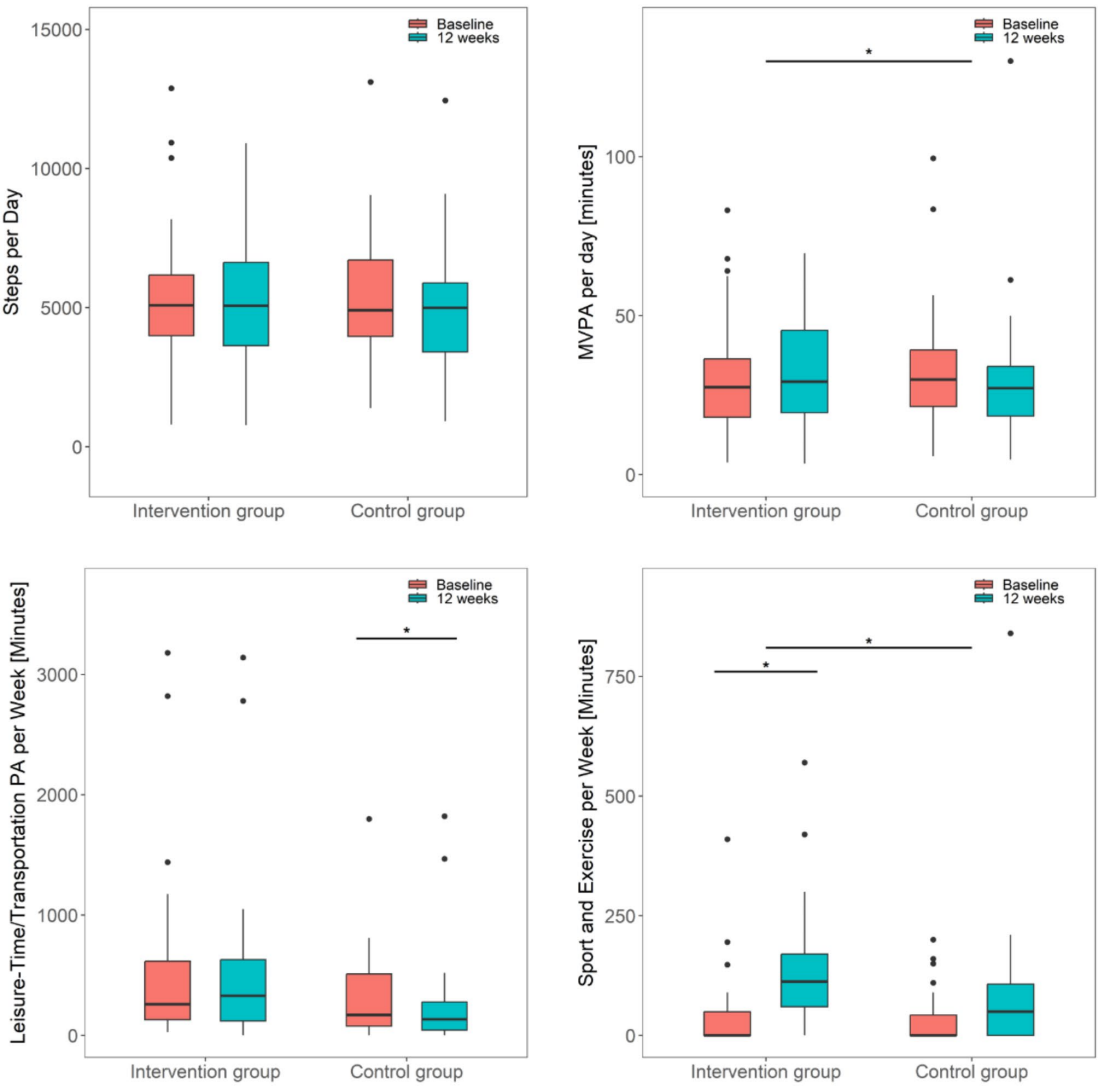
Boxplots of physical activity outcomes for both groups at baseline and after 12 weeks. Boxes: interquartile ranges; horizontal bar within the boxes: median values; whiskers: lowest and highest 25% of scores, maximum length: 1.5 times the interquartile range, dots: outliers

Results of the Mann-Whitney U tests are reported in Table 2. Changes in daily steps from baseline to the 12-week measurement did not differ significantly between groups (primary outcome). Significant differences were observed in the secondary outcomes device-measured MVPA minutes, and subjectively measured minutes of sport-/exercise-related activities. Minutes of sport- and exercise-related activities increased in both groups. These increases were significant in the IG (median_baseline_ = 0.0, median_12wks_ = 112.5, *p* =.005, d = 0.80) and almost significant in the CG (median_baseline_ = 0.0, median_12wks_ = 50.0, *p* =.053, d = 0.55) (Fig. 2). No significant differences were observed between groups for changes in minutes of leisure-time and transportation physical activity from baseline to the 12-week measurement. However, we observed a significant decrease in the CG (median_baseline_ = 170.0, median_12wks_ = 135.0; *p* =.029; d = 0.62).

The available case analysis confirmed results for daily steps as well as minutes of leisure-time and transportation physical activity. Device-measured MVPA minutes and self-reported minutes of sport-/exercise-related activity, were non-significant in the available case analysis (MVPA: n_IG_ = 22, n_CG_ = 25, median change_IG_= 0.3; median change_CG_ = -7.7, *p* =.134; sport and exercise: n_IG_ = 24, n_CG_ = 25, median change_IG_ = 88.8, median change_CG_ = 0.0; Z = 1.716, *p* =.088).

During the 12-week follow-up, the minutes of sport-/exercise-related activities decreased significantly (Table 3). However, the absolute level of sport-/exercise-related activities after the follow-up was still higher compared to baseline. Minutes of leisure-time and transportation physical activity increased significantly. Daily steps and MVPA minutes did not change significantly.

**Table 3:** Results of the intervention group for the follow-up period (T1-T2), and descriptive statistics

	*n*	Median_T0_ (IQR)	Median_T1_(IQR)	Median_T2_ (IQR)	*p*-value	ES (d)^b^
Steps per day	29	5086.4 (2179.9)	5072.4 (2991.0)	5134.5 (2406.7)	0.795	-0.07
MVPA [minutes/day]	29	27.5 (18.4)	29.3 (25.9)	27.4 (18.8)	0.110	-0.43
Leisure-time/transportation PA [minutes/week]	29	260.0 (485.9)	330.0 (510.0)	597.0 (576.9)	0.006	-0.78
Sport and exercise [minutes/week]	29	0.0 (49.6)	112.5 (110.0)	30.5 (110.0)	0.006	-0.77
Movement competence [%]	29	50.0 (22.5)	48.1 (31.7)	52.1 (26.6)	0.051	-0.53
Control competence [%]	29	39.9 (21.1)	51.8 (33.2)	55.9 (26.0)	0.624	-0.13
Self-regulation Competence [%]	29	64.8 (26.8)	69.1 (21.9)	67.8 (18.2)	0.332	-0.26
SSK-Index	21	3.3 (1.7)	3.0 (3.3)	3.0 (2.0)	0.490	-0.21
MSWS-12	18	54.2 (42.2)	34.4 (36.5)	41.7 (37.5)	0.501	-0.22
WEIMuS	20	41.50 (23.5)	32.00 (13.3)	34.00 (15.0)	0.212	-0.40
CES-D	15	17.0 (8.5)	17.0 (7.5)	20.0 (10.5)	0.683	-0.15
MSIS-29, physical subscale	21	36.3 (18.8)	27.5 (17.5)	30.0 (26.3)	0.032	-0.70
MSIS-29, psychological subscale	21	30.6 (24.3)	22.2 (22.2)	33.3 (33.3)	0.052	-0.63

Notes. Descriptives are reported as median and interquartile range. ^*b*^Cohen’s *d* (values around 0.2 represent small effects, values around 0.5 intermediate effects, and values around 0.8 strong effects). Abbreviations. 2MWT: Two-Minute Walk Test; CES-D: Center for Epidemiologic Studies Depression Scale; MSIS-29: Multiple Sclerosis Impact Scale; MSWS-12: Multiple Sclerosis Walking Scale; MVPA: moderate- to-vigorous intensity physical activity; SSK: sports-related self-concordance; T25FW: Timed 25-Foot Walk; WEIMuS: Würzburg Fatigue Inventory in MS

### Physical activity-related health competence

**Fig. 3:**
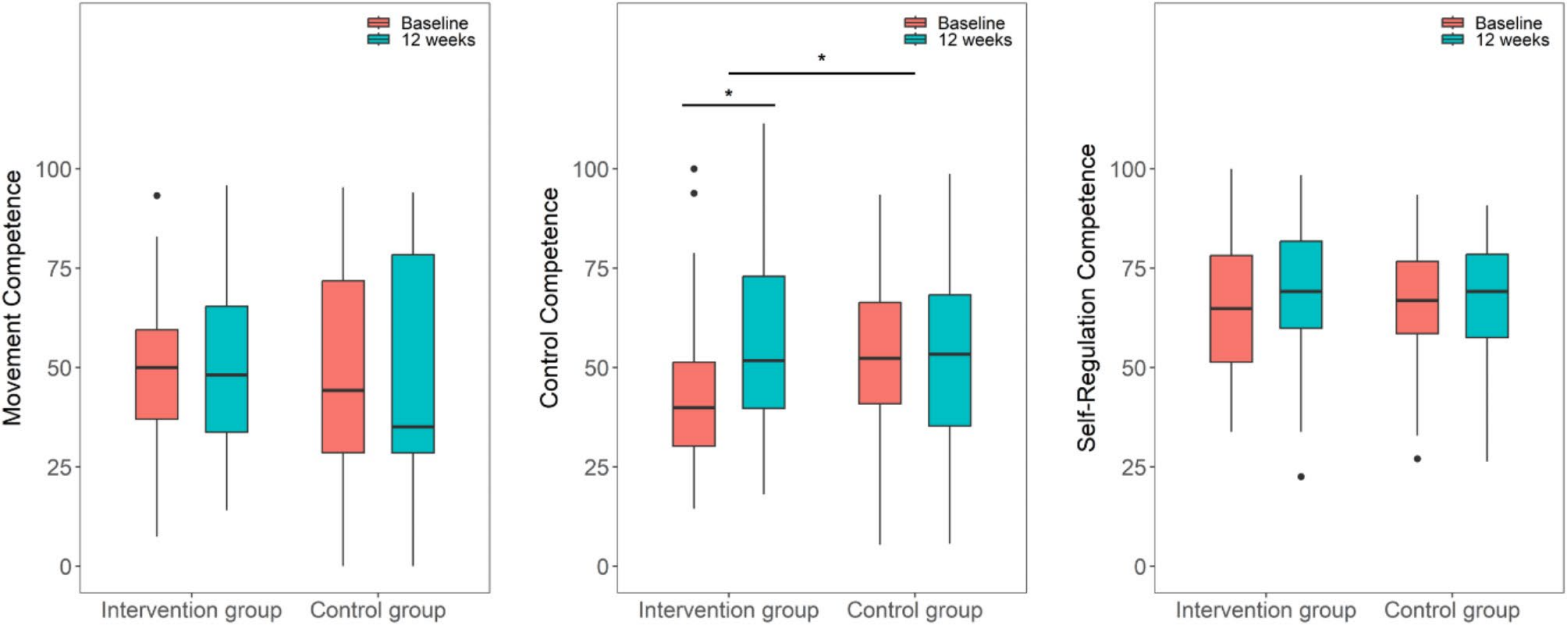
Boxplots of PAHCO subcompetences for both groups at baseline and after 12 weeks. Boxes: interquartile ranges; horizontal bar within the boxes: median values; whiskers: lowest and highest 25% of scores, maximum length: 1.5 times the interquartile range, dots: outliers

Changes from baseline to the 12-week measurement differed significantly between groups for control competence (Table 2). We observed a significant increase of control competence in the IG (median_baseline_ = 39.9, median_T12wks_ = 51.8; *p* =.001; d = 0.98) and no change of scores in the CG (median_baseline_ = 52.4, median_12wks_ = 53.3; *p* =.943; d = 0.02) (Fig. 3). No significant differences were observed between groups for changes of movement and self-regulation competence from baseline to the 12-week measurement. Over the follow-up period, movement and control competence increased, but changes did not reach significance level (Table 3). The available case sensitivity analysis confirmed results for the three subcompetences.

### Other outcomes

**Fig. 4:**
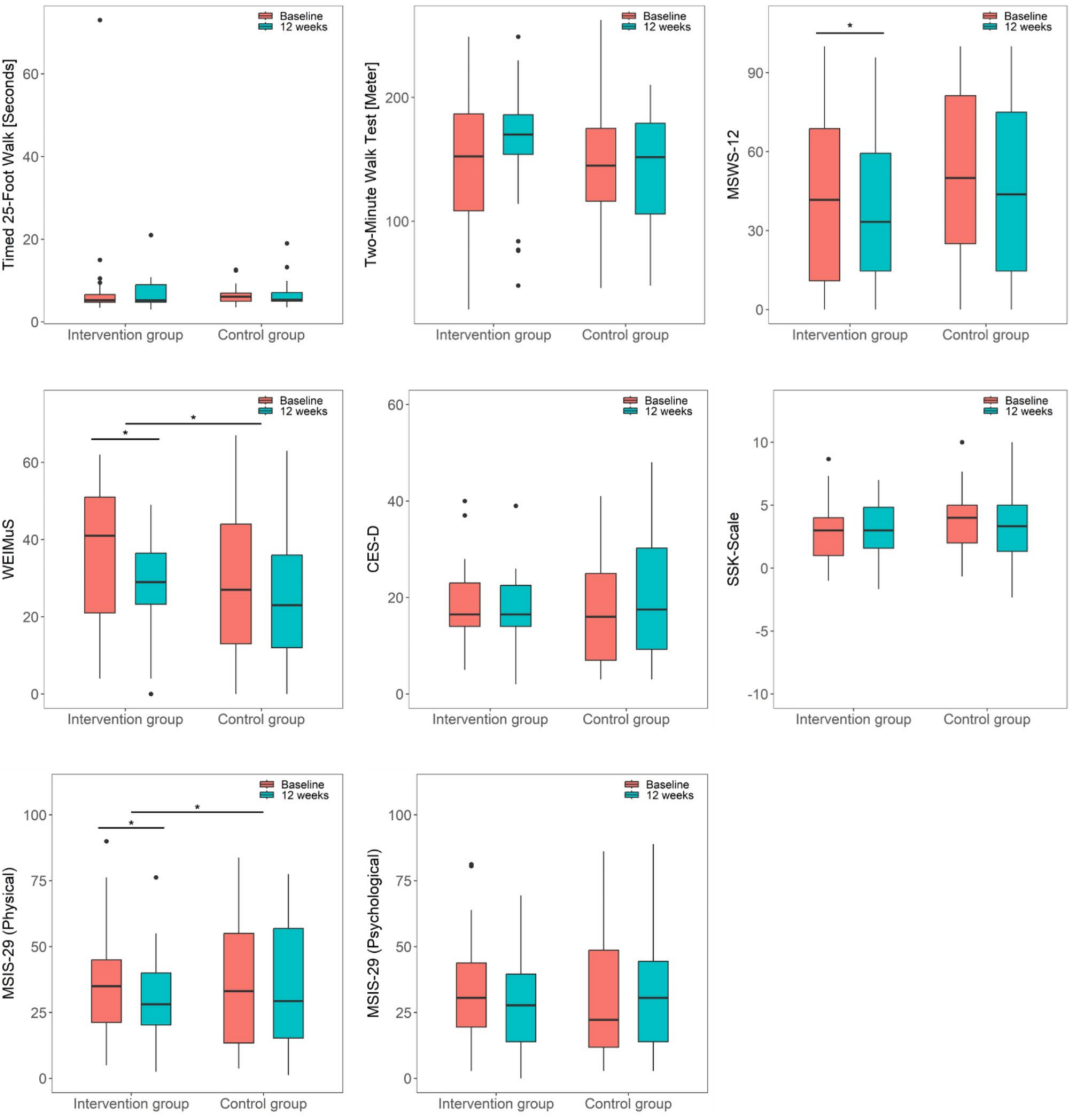
Boxplots of other outcome variables for both groups at baseline and after 12 weeks. CES-D: Center for Epidemiologic Studies Depression Scale; MSIS-29: Multiple Sclerosis Impact Scale; MSWS-12: Multiple Sclerosis Walking Scale; SSK: sports-related self-concordance; WEIMuS: Würzburg Fatigue Inventory in MS; Boxes: interquartile ranges; horizontal bar within the boxes: median values; whiskers: lowest and highest 25% of scores, maximum length: 1.5 times the interquartile range; dots: outliers

Changes in fatigue and the physical component of quality of life from the baseline to the 12-week measurement differed significantly between groups (Table 2). Fatigue decreased significantly in the IG (median_baseline_ = 41.0, median_12wks_ = 29.0; *p* =.002; d = 0.68) with no significant change in the CG (median_baseline_ = 25.0, median_12wks_ = 23.0; *p* =.229; d = 0.36) (Fig. 4). The physical subscale of quality of life improved significantly in the IG (median_baseline_ = 35.6, median_12wks_ = 28.1; *p* =.015; d = 0.75), while it did not change significantly in the CG (median_baseline_ = 35.0, median_12wks_ = 31.3; *p* =.733; d = 0.11). No significant differences between groups were observed for changes of the psychological component of quality of life, depression, PA-related and sports-related self-concordance, and walking ability measures (T25FW, 2MWT, MSWS-12) from baseline to the 12-week measurement.

During the 12-week follow-up, a significant reduction in quality of life was observed in both subscales. The remaining outcomes did not show significant changes (Table 3). The average ratings of usability and usefulness of the mobile application were 5.7 (SD = 1.0) and 5.6 (SD = 1.0).

Eleven adverse events were documented for the time from baseline to the 12-week measurement (9 in the IG). Five adverse events were documented for the time from the 12-week measurement to the 24-week measurement (all in the CG). The most common adverse events were falls (*n* = 2), relapses (*n* = 2), upper respiratory tract infections (*n* = 2), orthopedic issues (*n* = 2) and side effects of MS medication (*n* = 2).

### Intervention adherence and chat function usage in the app

Over the 12 weeks, participants completed on average 1.9 training sessions per week. The number of training sessions per week per participant declined through the course of the intervention (week 1: 2.2 sessions per week; week 12: 1.2 sessions per week). Across all participants, 68.5% of completed training sessions were resistance training, 28.9% endurance training and 2.6% with an unknown training modality. Participants completed on average 2.9 (SD = 4.2, minimum = 0, maximum = 13) out of 7.4 activated e-learning modules (SD = 3.9, minimum = 0, maximum = 13). 65.5% participants completed all four calls with the therapist, 20.7% completed three calls, 3.4% two calls, and 10.3% no calls. 17 out of 29 IG participants used the chat function of the app (58.6%). On average, 11.6 messages were exchanged between therapists and participants (SD = 10.8, minimum = 0, maximum = 36).

## Discussion

This randomized waitlist-controlled trial examined the effects of a 12-week internet-based exercise and PA promotion intervention in a sample of pwMS. No effects were observed for the primary outcome steps per day. However, we identified effects on some of the measured outcomes after 12 weeks, which are device-measured minutes of MVPA, self-reported minutes of sport and exercise, control competence, fatigue and the physical dimension of quality of life. During the 12-week follow-up period, effects on control competence and fatigue were maintained, minutes of leisure-time and transportation PA increased significantly, and minutes of sport and exercise as well as the physical dimension of quality of life decreased significantly.

A possible explanation for the absence of a positive intervention effect on daily steps is that our study participants may have increased PA types that cannot be adequately measured with daily steps. In line with the theoretical underpinnings of the PAHCO model and SDT, the developed intervention provided a resistance and endurance exercise plan, remotely supervised by trained exercise therapists, in conjunction with a behavioral intervention to promote unstructured PA with a focus on walking behavior and exercise in everyday life. The changes in device-measured minutes of MVPA and sport-/exercise-related activities, although significant only in the main analysis and not the available-case analysis, suggest that participants of the IG focused on exercising instead of increasing unstructured PA. Furthermore, our compliance data show that our intervention resulted in a more frequent execution of resistance training than endurance training. However, strengthening exercises cannot be adequately captured with accelerometers [63]. This may have contributed to missing changes in daily steps in the IG, despite an increase in overall PA. Conversely, all studies demonstrating significant within-group changes of daily steps administered a behavioral intervention that focuses on walking behavior without the additional provision of an exercise plan that is monitored by exercise therapists [19, 64, 65].

Looking at the median changes of PA outcomes from baseline to postintervention, both daily steps and minutes of leisure-time and transportation PA tend to decrease in both groups, although not significantly. It is important to note that the baseline data were collected from mid-October to the end of November, and the post-intervention data were collected from the end of January to the beginning of March. In the general population, shorter days, colder weather conditions and increased precipitation were identified as barriers for physical activity participation [66]. Furthermore, a recent review confirmed that physical activity levels are reduced during the winter season [67]. Studies that investigated seasonal effects on different intensities of PA found greater seasonal effects on low-intensity compared to moderate and high intensity PA [67]. Out of the PA outcomes used in this study, daily steps and leisure-time and transportation PA are the ones predominantly reflecting low-intensity activities that are performed outdoors. Thus, the seasonal effect of the winter could have contributed to the lack of effects in relation to leisure-time and transportation PA and the primary outcome steps per day.

During the follow-up period, minutes of leisure-time and transportation PA increased significantly in the IG, while minutes of sport and exercise decreased significantly. With regard to device-measured MVPA minutes, we also observed a non-significant trend towards a reduction. We assume that the absence of the personal therapists led to decreased exercise motivation and fewer training sessions during the follow-up phase. Also, the first COVID-lockdown made the transition to other structured exercise programs more difficult. The intervention effects on fatigue and control competence may have facilitated an increase in leisure-time and transportation PA, which had already started during the intervention period and reached significance during the follow-up. Furthermore, the seasonal change from winter to spring could have contributed to an increase low-intensity PA [67], and consequently to an increase in daily steps and minutes of leisure-time and transportation PA.

Furthermore, the 12-week intervention period had a positive effect on one of the subcompetences of PAHCO, which is control competence. Increases in control competence suggest that participants increased their ability to direct their PA towards maintaining and promoting health and well-being through the intervention. This may have contributed to observed effects on fatigue and quality of life. Movement competence did not change during the intervention and follow-up periods. As mentioned earlier, we did not prescribe a rigid exercise regimen with the aim to have maximal effects on physical performance and with this on movement competence. Instead, participants set personalized PA goals with their therapists and were prompted to reflect their PA goals, plans, and behavior regularly in order to increase self-regulation competence. Thus, the stimulus of executed PA may have been inadequate to cause effects on movement competence in our sample within the 12-week intervention. However, the increased PA levels and control competence that were present after the follow-up period may cause effects on movement competence in the long-term. We could already observe improvements in movement competence during the follow-up period that almost reached statistical significance. Longer follow-ups would have been needed to control whether changes in control competence and PA convert into effects on movement competence. Interestingly, although self-regulation competence did not increase in this study, participants were able to increase their volume of leisure-time and transportation PA during the follow-up period.

Current reviews support the effects of exercise on walking ability, fatigue, depression and quality of life in pwMS [5–7, 11]. However, only a few internet-based behavioral interventions were able to improve some of these symptoms of pwMS. These interventions were either 6-month behavioral interventions based on social cognitive theory [19, 56, 68] or 12-week rehabilitation aftercare programs for pwMS suffering from fatigue [69]. We achieved effects on fatigue and the physical dimension of quality of life with a 12-week behavioral intervention for pwMS. Against the backdrop that fatigue is a common symptom of MS that is often untreated [70], such an intervention can complement current MS care. However, effects on fatigue need to be interpreted cautiously and need to be confirmed in future studies. At baseline, fatigue levels in the IG were higher than in the CG. Even though this difference was non-significant, it might have been clinically relevant and could have influenced observed differences in fatigue reduction between groups.

With an average training frequency of 1.9 times per week, the minimum recommended training frequency in this study was almost met. Training compliance is comparable to our previous studies examining internet-based exercise training and behavioral interventions for pwMS [69, 71]. In line with these studies, we observed a decline in training sessions documented in the study app over the 12-week intervention period. However, we do not know to what extent this decline is attributable to lower training adherence or reduced documentation compliance. If the training frequency of 1.9 times per week reflects the true training frequency, the frequency might have been high enough to increase aspects of PA as well as control competence within 12-weeks but not high enough to achieve significant changes in some of the other outcomes measured like walking ability, depression and the psychological component of quality of life.

The majority of participants completed all calls with their therapists, which indicates that these calls were well received by the participants. With an average of 2.9 completed modules out of 7.4 activated module per participants, completion rates of e-learning modules were lower than expected. The access of e-learning modules through a platform other than the study app may have been a barrier for module consumption. Integrating the content from e-learning modules in the study app, may increase consumption of modules and with this increase effects on PAHCO and PA.

Our study has some limitations. We included only ambulatory pwMS and most study participants were female and had RRMS. Thus, study results may not be transferable to alle pwMS. The study did not reach the planned sample size, and thus, not the planned statistical power. The reduced power results in a lower probability to discover a true effect, a lower chance that a significant effect reflects a true effect and a potential overestimation of effect sizes [72]. Also, due to the low sample size, we did not correct for multiple testing in the secondary outcomes. Thus, the secondary analyses should be regarded as exploratory. Mean imputation of missing values, which was used in the main analysis, is not regarded as the gold standard to replace missing values, since it reduces variability and distorts distribution of variables [62]. This may have influenced the results of the main analysis. The absence of a CG during the follow-up period hampers the interpretation of changes during that phase. In addition, the follow-up period coincided with the COVID-19 lockdown in Germany. On 22nd March 2020, several measures came into force that restricted social contacts comprehensively in Germany [73]. Restrictions were gradually loosened and major contact restrictions lifted in June 2020 [73]. The COVID-19 pandemic seemed to decrease overall PA levels in pwMS [74, 75]. An explorative analysis of daily steps recorded with consumer activity-monitors in our study from November 2019 until end of April 2020 revealed a significant decrease of daily steps from the week of first restrictions to (calendar week 12) to calendar week 15 [76]. Furthermore, the COVID-19 lockdown may have worsened symptom levels [77, 78], and new environmental conditions asked for adaptations in PA behavior [79, 80]. Thus, negative effects of the lockdown on measured PA outcomes and symptoms in our study sample during the follow-up period are likely. Also, only five out of eight study centers provided information on the number of patients screened, which limits conclusions on the generalizability of the study results. In those study centers that provided data, about 73% of the screened patients were enrolled in the study. Furthermore, we recruited our study sample via neurological practices, hospitals and rehabilitation centers. In combination with our broad inclusion criteria, this led to a rather heterogeneous study sample. This sample, in turn, received a complex, personalized intervention. In practice, this resulted in a large amount of intervention variants consisting of several intervention components. This makes it difficult to identify effective intervention components or the optimal dosage. To improve understanding of study results and effective intervention components, a study with a bigger sample size, allowing for subgroup or moderator analyses, as well as a detailed process evaluation is necessary.

## Conclusions

Our study provides first evidence that the developed 12-week internet-based exercise and PA promotion intervention can increase health-enhancing PA in pwMS. Even though we could not show positive effects on the primary outcome (device-measured steps per day), we found intervention effects on control competence, device-measured MVPA minutes, minutes of self-reported sport and exercise, fatigue and quality of life. Furthermore, participants significantly increased their minutes of leisure-time and transportation PA during the follow-up, and effects on control competence and fatigue could be maintained. This suggests that this intervention, with its competence-oriented and personalized approach, is able to improve PAHCO and through this aspects of PA and health in pwMS. With this approach, the internet-based intervention is highly versatile and has great potential to complement on-site MS care and increase PA levels of pwMS. Future research needs to confirm our results in trials with larger sample sizes. These trials would allow for subgroup analyses to improve understanding of intervention mechanism.

## Electronic supplementary material

Below is the link to the electronic supplementary material.

Supplementary Material 1

Supplementary Material 2

## Data Availability

The datasets used and analyzed during the current study are available from the corresponding author on reasonable request.

## Abbreviations

2MWT: Two-Minute Walk Test
ANOVA: Analysis of variance
CES-D: Center for Epidemiologic Studies Depression Scale
CG: Control group
EDSS: Expanded Disability Status Scale
IG: Intervention group
MS: Multiple sclerosis
MSIS-29: Multiple Sclerosis Impact Scale
MSWS-12: Multiple Sclerosis Walking Scale
MVPA: Moderate-to-vigorous intensity physical activity
pwMS: Persons with multiple sclerosis
PA: Physical activity
PAHCO: Physical activity-related health competence
RPE: Rate of perceived exertion
SD: Standard deviation
SDT: Self-determination theory
T25FW: Timed 25-Foot Walk
WEIMuS: Würzburg Fatigue Inventory in MS
