# Supplementary material for: Internet-based exercise and physical activity promotion for persons with multiple sclerosis: a randomized controlled trial
Source: BMC Sports Sci Med Rehabil. 2025 Apr 23;17:90. doi: 10.1186/s13102-025-01146-x (PMC12016365; doi:10.1186/s13102-025-01146-x)
Supplement: Supplementary file 1 — Supplementary Material 1 [file 13102_2025_1146_MOESM1_ESM.docx]

**Supplement 1**

Table S1. Overview of the intervention content that was used to promote the three subcompetences of the PAHCO model and associated behavior change techniques.

| PAHCO Subcompetence | Intervention Content | Associated BCT according to Michie et al.*^a^* |
| --- | --- | --- |
| Movement Competence | - Prompt regular progressive endurance, resistance and coordination training prescribed by certified therapists - Advise participants on how to execute each exercise through movement descriptions and pictures (e-learning, app) - Provide information about basic body positions for resistance exercises through text and pictures (e-learning) - Prompt to perform body perception exercises to improve lumbar spine position during resistance training (e-learning) - Prompt reflection of perceived physical exertion after each exercise to improve body awareness (app) | 8.1 Behavioral practice/ rehearsal  4.1 Instructions on how to perform a behavior  6.1 Demonstration of the behavior  9.1 Credible source |
| Control Competence | - Provide information about effects of PA and exercise on health and MS specific symptoms (e-learning) - Provide information about risks and safety of PA and exercise for persons with MS (Fatigue, Uhthoff) (e-learning) - Provide information about the optimal Dose of PA and exercise based on MS specific recommendations (e-learning) - Provide information about main training principles, basics of strength, endurance and balance training and special considerations for pwMS (e-learning) - Prompt reflection of proportion between own exercise/PA and recovery phases (e-learning) - Provide information on how to determine and control training intensity using rate of perceived exertion, heart rate, and respiratory rate (e-learning) - Prompt application of methods to determine and control exercise intensity (e-learning, app) (exercise in e-learning and continuous application during training) | 5.1. Information about health consequences  5.6. Information about emotional consequences  4.1 Instruction on how to perform a behavior (training control)  4.2 Information about antecedents  9.1 Credible source |
| Self-Regulation Competence | - Participants agree on a behavioral goal and plan with therapist (steps per day and training) and document them as personal goals in the app (1:1 call) - Provide information about SMART goals (e-learning) and prompt reflection of quality of own goals (e-learning) - Prompt development of personal SMART goals (e-learning) - Provide Information about action planning (Importance and description on how to develop good plans (e-learning) - Encourage planning personal PA at a particular time, on certain days of the week and a specific place (1:1 call, e-learning) - Provide information about common general and MS-specific barriers of and strategies to counteract them (e-learning) - Prompt identification of personal barriers of PA and development of personal strategies to counteract them (e-learning) - Prompt identification of facilitating factors (1:1 call) - Provide information about self-monitoring material and techniques (e.g. PA diary, activity monitors) (e-learning) - Documentation of own PA behavior and objective measurement of PA behavior and training load development (activity monitors, training documentation and PA diaries in app) - Regular reflection of PA behavior and comparison to personal goals and action planning (e-learning, group video calls, 1:1 calls with therapist)/Examine how well behavioral goals were met and adapting goals and plans if discrepancies are present (1:1 call, e-learning) - Provide information about motives for PA and exercise and prompt identification of own motive(s) for PA/exercise (e-learning) - Motivational Interviewing principles applied by therapists during calls - Gradual progression of training load (repetitions, sets, duration of endurance exercises) based on participant's capacity (app) - Prompt reflection of training effects and other positive experiences with PA and exercise (Group video calls) - Agreement on personalized behavior goals, regular reflection and adaptation if needed/possible (1:1 calls, group video calls). - Prescription of lower exercise intensity to familiarize with training at the beginning and increase of exercise intensity in the second half of the program (app) | 1.1 Goal setting (behavior)  1.2 Problem Solving  1.4. Action planning  1.5 Review behavior goals  1.6 Discrepancy between current behavior and goal  1.8 Behavioral contract  2.3 Self-monitoring of behavior  2.4 Self-monitoring of outcomes of behavior  4.2 Information about antecedents  8.7 Graded tasks  9.1 Credible Source |

**Abbreviations.** BCT = behavior change technique, MS = multiple sclerosis, PA = physical activity, pwMS = persons with multiple sclerosis. **References.** Michie S, Richardson M, Johnston M, Abraham C, Francis J, Hardeman W, et al. The behavior change technique taxonomy (v1) of 93 hierarchically clustered techniques: building an international consensus for the reporting of behavior change interventions. Ann Behav Med. 2013;46:81–95. doi:10.1007/s12160-013-9486-6.

**Supplement 2**

|  | **Key component techniques for need support as described by Silva et al. 2014*^a^*** | **Implementation of the key component techniques for need support*^a^* in the MS bewegt program** |
| --- | --- | --- |
| Autonomy support | - Relevance, by providing a clear and meaningful rationale for activities, facilitating self-endorsement - Respect, by acknowledging the importance of clients’ perspective, feelings, and agenda - Choice, by encouraging clients to follow their own interests and providing options whenever possible - Avoidance of control, by not using coercive, authoritarian, or guilt-inducing language or methods. | - Clear communication of the rationale of all intervention components including why telephone and video meetings are performed, why e-Learning modules are offered, which goals the single modules have, why exercise and physical activity are important and why using the activity trackers might be beneficial. - Intervention goals were set individually with the patients acknowledging their personal goals and resources. - Participants were able to communicate exercise preferences and also exercises they do not like. The exercise plans were created and adapted accordingly. - Therapists’ communication strategies during chats, telephone and group video calls were informed by motivational interviewing*^b^* |
| Support for competence | - Clarity of expectations, by collaboratively setting realistic goals and discussing what to expect and not expect from the behavior-linked outcomes - Optimal challenge, by tailoring strategies and goals to individuals´ skills - feedback, offering clear and relevant informational feedback (e.g. on goal progress), in a non-judgmental manner - Provision of instrumental and practical skills-training, guidance, and support | - Goals were set collaboratively at the beginning of the intervention and were reflected and during each telephone and video meeting. - Behavioral goals and exercise plans were tailored to each participant’s motor abilities and skills as well as experiences symptoms of MS. Information about individual prerequisites was collected by therapists at the beginning of the intervention. Participants were able to provide feedback regarding the level of difficulty and the perceived intensity at the end of each exercise session. - Regular feedback based on training and learning modules was provided during telephone and video calls (which were informed by motivational interviewing*^b^)* - Skill-training, guidance and support was provided in order to improve physical activity-related health competence (see supplement 1) |
| Support for relatedness | - Empathy, by attempting to see the situation through the client’s perspective - Affection, by displaying genuine appreciation and concern for the person - Attunement, through paying careful attention to and gathering knowledge about the person - Dedication of resources, through volunteering time and energy - Dependability, through availability in case of need | - Therapists’ communication strategies during chats, telephone and group video calls were informed by motivational interviewing*^b^* - At the beginning of the intervention, therapists gathered all information needed to create a tailored exercise plan. - Therapists closely monitored exercise activities and contacted participants if feedback indicated any problems. - Training plans were progressed based on participants’ feedback in the study app and during telephone and video meeting. - Participants were able to contact therapists through the chat function in the study app. |

*^a^* Silva, M. N., Marques, M., & Teixeira, P. J. (2014). Testing theory in practice: The example of self-determination theory-based interventions. The European Health Psychologist, 16(5), 171–180.

*^b^* Miller WR, Rollnick S. Motivational interviewing: Helping people change and grow. 4th ed. New York: Guilford Press; 2023.

**Supplement 3**

Table S2. E-learning modules of the developed program.

| **Subject Area** | **Module** |
| --- | --- |
| Technology in MS bewegt | Step 1: Install the MS bewegt app |
|  | Step 2: Get to know the MS bewegt app |
|  | Step 3: Connect the app with your activity monitor |
|  | Step 4: Prepare for the video call with your therapist |
| Exercise and symptoms of MS | Exercising in spite of and with fatigue |
|  | Exercising with heat sensitivity |
|  | Exercising and walking ability |
| Volitional and motivational prerequisites of physical activity | Why do I want to be physically active? |
|  | Goal setting |
|  | Planning physical activity |
|  | Overcoming barriers |
|  | Monitor yourself |
| How to plan and monitor exercise | Effects of sports and physical activity |
|  | How much exercise is recommended? |
|  | Training principles - an introduction |
|  | Load control in endurance training |
|  | Load control in exercise training |

**Abbreviations.** MS = multiple sclerosis

**Supplement 4**

Table S4. Median and mean differences between baseline (T0) and after 12 weeks (T1) for all available cases and results of the Mann-Whitney-U-Test.

| **Dependent variables** | **Intervention group** | | | **Control Group** | | | **Test-Statistics** | | |  |
| --- | --- | --- | --- | --- | --- | --- | --- | --- | --- | --- |
|  | **n** | **Median change_T1-T0_ (IQR)** | **Mean**  **change_T1-T0_**  **(SD)** | **n** | **Median**  **change_T1-T0_**  **(IQR)** | **Mean**  **change_T1-T0_ (SD)** | | **p** | **ES (d*^a^*)** | |
| Steps per day | 23 | -153.9  (1499.2) | -16.5  (1474.0) | 25 | -552.7  (1404.5) | -431.8  (1425.2) | | 0.268 | 0.32 | |
| MVPA [minutes/day] | 22 | 0.3  (14.3) | 1.8  (15.0) | 25 | -7.7  (12.8) | -3.7  (13.3) | | 0.134 | 0.45 | |
| Leisure-time/transportation PA [minutes/week] | 16 | -10.0  (242.50) | -39.1  (245.9) | 16 | -42.5  (354.6) | -42.9  (632.5) | | 0.559 | 0.21 | |
| Sport and exercise [minutes/week] | 24 | 88.8  (136.3) | 98.5  (173.3) | 25 | 0.0  (60.0) | 54.7  (178.4) | | 0.088 | 0.50 | |
| Movement competence [%] | 24 | -0.3  (16.8) | -0.3  (15.3) | 26 | -1.5  (10.0) | -0.9  (8.7) | | 0.634 | 0.13 | |
| Control competence [%] | 24 | 12.5  (22.5) | 11.4  (17.7) | 26 | 0.5  (16.8) | 0.3  (15.5) | | 0.011 | 0.77 | |
| Self-regulation Competence [%] | 24 | 2.5  (16.3) | 3.8  (15.0) | 26 | 0.3  (14.6) | -0.7  (12.5) | | 0.449 | 0.22 | |

**Notes.** *^a^*Cohen’s *d* (values around 0.2 represent small effects, values around 0.5 intermediate effects, and values around 0.8 strong effects). **Abbreviations:** IQR: interquartile range; MVPA: moderate-to-vigorous intensity physical activity; PA: physical activity; SD: standard deviation

**Supplement 5**

Table S3. Median and interquartile ranges at baseline (T0) and 12 weeks (T1) in the intervention and control group and results of the Wilcoxon signed-rank tests.

| **Dependent variables** | **Intervention group** | | | | | **Control group** | | | | |
| --- | --- | --- | --- | --- | --- | --- | --- | --- | --- | --- |
|  | **n** | **T0** | **T1** | **p** | **ES (d*^a^*)** | **n** | **T0** | **T1** | **p** | **ES (d*^a^*)** |
| Steps per day | 29 | 5086.4 (2179.9) | 5072.4 (2991.0) | .966 | 0.01 | 27 | 4907.3 (2744.4) | 4996.3 (2484.8) | .056 | 0.54 |
| MVPA [minutes/day] | 29 | 27.5  (18.4) | 29.3  (25.9) | .173 | 0.36 | 27 | 29.9  (17.8) | 27.4  (15.7) | .070 | 0.51 |
| Leisure-time/transportation PA [minutes/week] | 29 | 260.0  (485.9) | 330.0  (510.0) | .194 | 0.35 | 27 | 170.0  (432.3) | 135.0  (232.5) | .029 | 0.62 |
| Sport and exercise [minutes/week] | 29 | 0.0  (49.6) | 112.5  (110.0) | .005 | 0.80 | 27 | 0.0  (42.5) | 50.0  (107.4) | .053 | 0.55 |
| Movement competence [%] | 29 | 50.0  (22.5) | 48.1  (31.7) | .966 | 0.01 | 27 | 44.2  (43.3) | 35.1  (49.9) | .648 | 0.12 |
| Control competence [%] | 29 | 39.9  (21.1) | 51.8  (32.2) | .001 | 0.98 | 27 | 52.4  (25.6) | 53.3  (33.0) | .943 | 0.02 |
| Self-regulation Competence [%] | 29 | 64.8  (26.8) | 69.1  (21.9) | .103 | .044 | 27 | 66.9  (18.1) | 69.1  (21.0) | .960 | 0.01 |
| SSK-Index | 24 | 3.0  (2.17) | 3.0  (3.3) | .961 | 0.01 | 26 | 4.0  (3.2) | 3.3  (3.7) | .010 | 0.77 |
| 2MWT | 20 | 150.2  (78.9) | 169.0  (42.5) | .260 | 0.36 | 23 | 145.0  (59.4) | 151.8  (73.3) | .715 | 0.11 |
| T25FW | 20 | 5.4  (2.6) | 5.1  (2.9) | .067 | 0.60 | 23 | 6.1  (2.0) | 5.4  (2.1) | .846 | 0.06 |
| MSWS-12 | 23 | 54.2  (55.2) | 33.3  (44.8) | .033 | 0.66 | 25 | 50.0  (58.3) | 43.8  (60.4) | .100 | 0.48 |
| WEIMuS | 22 | 41.0  (26.3) | 29.0  (13.3) | .002 | .0.68 | 23 | 25.0  (30.5) | 23.0  (26.5) | .229 | 0.36 |
| CES-D | 17 | 17.0  (8.0) | 17.0  (9.0) | .568 | 0.20 | 20 | 16.5  (18.3) | 17.5  (23.5) | .628 | 0.15 |
| MSIS-29, physical subscale | 24 | 35.6  (20.9) | 28.1  (19.7) | .015 | 0.75 | 23 | 35.0  (43.1) | 31.3  (43.1) | .733 | 0.11 |
| MSIS-29, psychological subscale | 24 | 31.9  (25.0) | 27.8  (25.7) | .140 | 0.44 | 23 | 22.2  (34.7) | 30.6  (30.6) | .627 | 0.15 |

**Notes.** Descriptives for T0 and T1 are reported as median and interquartile range, *^a^*Cohen’s *d* (values around 0.2 represent small effects, values around 0.5 intermediate effects, and values around 0.8 strong effects). **Abbreviations:** 2MWT: Two-Minute Walk Test; CES-D: Center for Epidemiologic Studies Depression Scale; MSIS-29: Multiple Sclerosis Impact Scale; MSWS-12: Multiple Sclerosis Walking Scale; MVPA: moderate-to-vigorous physical activity; PA: physical activity; SSK: sports-related self-concordance; T25FW: Timed 25-Foot Walk; WEIMuS: Würzburg Fatigue Inventory in Multiple Sclerosis
